# TLR4-mediated inflammation promotes foam cell formation of vascular smooth muscle cell by upregulating ACAT1 expression

**DOI:** 10.1038/cddis.2014.535

**Published:** 2014-12-18

**Authors:** Y-W Yin, S-Q Liao, M-J Zhang, Y Liu, B-H Li, Y Zhou, L Chen, C-Y Gao, J-C Li, L-L Zhang

**Affiliations:** 1 Department of Neurology, Institute of Surgery Research, Daping Hospital, Third Military Medical University, 10 Changjiang Branch Road, Yuzhong District, Chongqing 400042, China

## Abstract

Vascular smooth muscle cell (VSMC) foam cell formation is an important hallmark, especially in advanced atherosclerosis lesions. Acyl-coenzyme A:cholesterol acyltransferase 1 (ACAT1) promotes foam cell formation by promoting intracellular cholesteryl ester synthesis. The present study tests the hypothesis that oxidized low-density lipoprotein (oxLDL) increases the ACAT1 expression by activating the Toll-like receptor 4 (TLR4)-mediated inflammation, and ultimately promotes VSMC foam cell formation. Wild-type, ApoE^−/−^, TLR4^−/−^ and ACAT1^−/−^ mice on a C57BL/6J background were used. Increased TLR4, proinflammatory cytokines and ACAT1 were observed in high-fat (HF) diet-induced atherosclerotic plaque formation and in oxLDL-stimulated VSMCs. ACAT1 deficiency impeded the HF diet-induced atherosclerotic plaque formation and impaired the TLR4-manipulated VSMC foam cell formation in response to oxLDL. TLR4 deficiency inhibited the upregulation of myeloid-differentiating factor 88 (MyD88), nuclear factor-*κ*B (NF-*κ*B), proinflammatory cytokines and ACAT1, and eventually attenuated the HF diet-induced atherosclerotic plaque formation and suppressed the oxLDL-induced VSMC foam cell formation. Knockdown of MyD88 and NF-*κ*B, respectively, impaired the TLR4-manipulated VSMC foam cell formation in response to oxLDL. Rosiglitazone (RSG) attenuated HF diet-induced atherosclerotic plaque formation in ApoE^−/−^ mice, accompanied by reduced expression of TLR4, proinflammatory cytokines and ACAT1 accordingly. Activation of peroxisome proliferator-activated receptor *γ* (PPAR*γ*) suppressed oxLDL-induced VSMC foam cell formation and inhibited the expression of TLR4, MyD88, NF-*κ*B, proinflammatory cytokines and ACAT1, whereas inhibition of PPAR*γ* exerted the opposite effect. TLR4^−/−^ mice and VSMCs showed impaired atherosclerotic plaque formation and foam cell formation, and displayed no response to PPAR*γ* manipulation. In conclusion, our data showed that oxLDL stimulation can activate the TLR4/MyD88/NF-*κ*B inflammatory signaling pathway in VSMCs, which in turn upregulates the ACAT1 expression and finally promotes VSMC foam cell formation.

Atherosclerosis remains the major cause of deaths worldwide, with deteriorated clinical consequence of cardiovascular diseases including myocardial infarction and stroke.^1^ In 2008, for example, 17.3 million deaths were caused by cardiovascular diseases, and this number will increase to 23.3 million by 2030.^2^ Therefore, a better understanding of mechanisms involved in atherosclerosis may advance the development of comprehensive therapeutic regimens.

Foam cell formation from macrophages or vascular smooth muscle cells (VSMCs) is a crucial event in the development of atherosclerosis. Acyl-coenzyme A:cholesterol acyltransferase 1 (ACAT1) is an intracellular enzyme that converts free cholesterol into cholesteryl esters for storage in lipid droplets, and promotes foam cell formation in atherosclerotic lesions.^3, 4, 5^ ACAT1 activity is present in a variety of cells and tissues, including the macrophages, neurons, cardiomyocytes, VSMCs, mesothelial cells, alveolar and intestinal epithelial cells and hepatocytes.^6^ In macrophages, the involvement of ACAT1 in foam cell formation has been demonstrated by studies, and multiple molecular mechanisms have been put forward. A well-accepted mechanism is that inflammation increases the expression of ACAT1, promotes the intracellular lipid accumulation and ultimately leads to foam cell formation.^7^ However, in contrast, the mechanisms underlying VSMC foam cell formation, especially the role of ACAT1 in this process, remain largely unelucidated.

It is widely accepted that atherosclerosis involves chronic inflammatory reaction.^8^ Toll-like receptor 4 (TLR4), one intensively investigated member of the TLR family, has a critical role in initiating inflammation, and participates in VSMC activation.^9, 10^ Lipopolysaccharide (LPS) is a TLR4-specific ligand that can trigger TLR4-mediated inflammation. A previous study showed that *Chlamydia pneumoniae*, which contains LPS in its outer membrane, promotes low-density lipoprotein-induced macrophage-derived foam cell formation via upregulation of the expression of ACAT1.^11^ This further enhanced the association between inflammation and intracellular lipid disorder. However, considering that VSMCs in normal conditions do not have inflammatory properties similar to macrophages, it is unclear whether the TLR4-mediated inflammatory mechanism is also involved in the regulation of ACAT1 in VSMC foam cell formation. Herein, the present study tests the hypothesis that oxidized low-density lipoprotein (oxLDL) increases the ACAT1 expression by activating the TLR4-mediated inflammation, and ultimately promotes VSMC foam cell formation.

## Results

### ACAT1 has a critical role in atherosclerotic plaque formation and in oxLDL-induced VSMC foam cell formation

**Figure 1:**
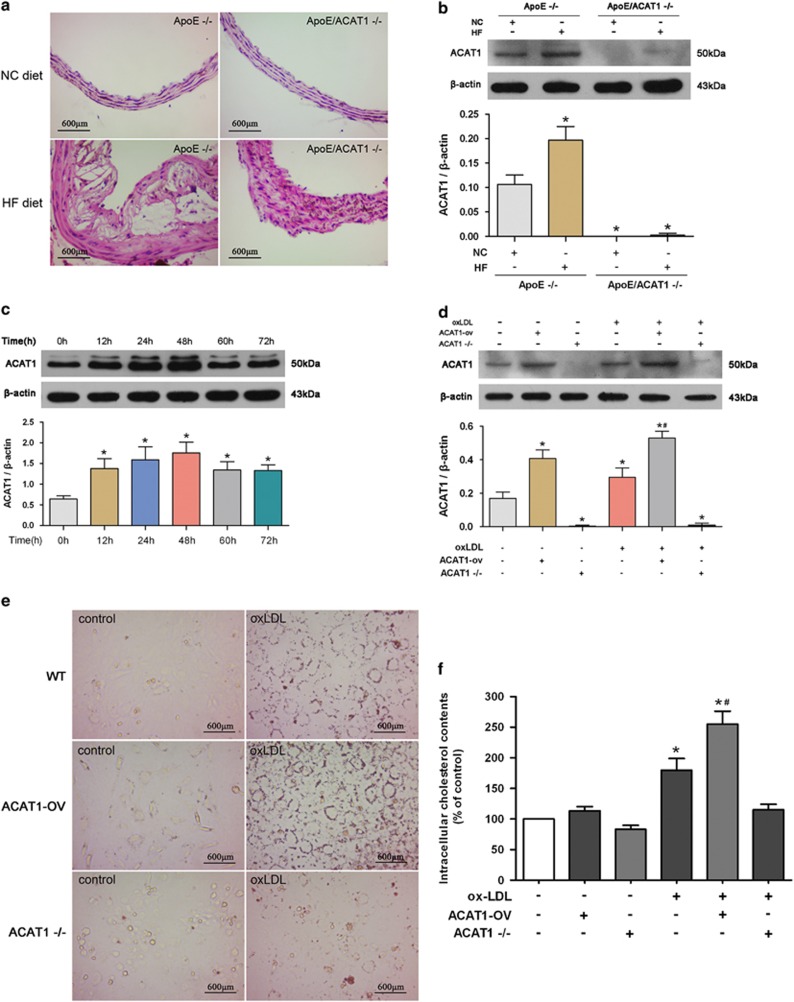
ACAT1 has a critical role in atherosclerotic plaque formation and in oxLDL-induced VSMC foam cell formation. (**a**) Hematoxylin and eosin staining on cross-sections from representative aortas are presented. HF diet significantly induced atherosclerotic plaque formation in ApoE^−/−^ mice. ApoE/ACAT1^−/−^ mice only displayed intimal hyperplasia in response to HF diet. (**b**) ACAT1 expression in aortas detected by western blot. HF diet induced ACAT1 expression in ApoE^−/−^ mice but not in ApoE/ACAT1^−/−^ mice (*P<0.05 *versus* ApoE^−/−^ mice with NC diet). (**c**) Primary VSMCs from WT mice were incubated with oxLDL (80 *μ*g/ml) for different times (0, 12, 24, 48, 60 or 72 h). ACAT1 level was increased in a time-dependent manner, with an obvious effect at 48 h after oxLDL challenge (*P<0.05 *versus* 0 h). (**d**–**f**) Primary VSMCs from WT mice were manipulated with adenovirus-mediated overexpression (ACAT1-ov) or knockout-mediated gene deficiency (ACAT1^−/−^) and then treated with oxLDL for 24 h (**d**). Cultured VSMCs in basal conditions displayed low levels of lipid droplet accumulation (**e**) and intracellular cholesterol (**f**), which were significantly elevated by oxLDL. ACAT1 overexpression increased, whereas ACAT1 deficiency reduced, the oxLDL-induced lipid droplet accumulation (**e**) and intracellular cholesterol elevation (**f**) (*P<0.05 *versus* control WT-VSMCs; ^#^*P*<0.05 *versus* WT-VSMCs with oxLDL challenge). Results were presented as mean±S.D. (error bars) of three independent experiments

To test the role of ACAT1 in atherosclerotic plaque formation, ApoE knockout (ApoE^−/−^) mice and ApoE/ACAT1 double-knockout (ApoE/ACAT1^−/−^) mice were used and fed with a high-fat (HF) diet. As shown in Figure 1a, HF diet elicited significant formation of atherosclerotic plaque in the aortas of ApoE^−/−^ mice, identified by hematoxylin and eosin staining. In contrast, ApoE/ACAT1^−/−^ mice only displayed intimal hyperplasia in response to HF diet. Besides, HF diet markedly increased the expression of ACAT1 in ApoE^−/−^ mice, whereas ApoE/ACAT1^−/−^ mice exhibited undetectable expression of ACAT1 in the aortas (Figure 1b). These data indicate an important role for ACAT1 in HF diet-induced atherosclerotic plaque formation.

The role of ACAT1 in VSMC foam cell formation was tested *in vitro*. We first detected the expression of ACAT1 in oxLDL-treated VSMCs. As shown in Figure 1c, oxLDL upregulated ACAT1 expression in a time-dependent manner, with an obvious effect at 48 h. Subsequently, the ACAT1 expression decreased slightly and tended to be stabilized. Next, we used gene knockout and adenovirus-mediated overexpression to manipulate the expression of ACAT1 (Figure 1d). The impact of ACAT1 on VSMC foam cell formation was subsequently detected. As shown in Figures 1e and f, in response to oxLDL challenge, VSMCs showed increased lipid droplets in the cytoplasm stained with Oil Red O, and also the intracellular cholesterol level increased markedly. ACAT1 overexpression further promoted, whereas ACAT1 deficiency markedly inhibited, the oxLDL-induced lipid droplet accumulation and intracellular cholesterol elevation, and thus affected the VSMC foam cell formation, indicating that ACAT1 has a critical role in foam cell formation in oxLDL-treated VSMCs.

### TLR4-mediated inflammation is required in atherosclerotic plaque formation and in oxLDL-induced VSMC foam cell formation

**Figure 2:**
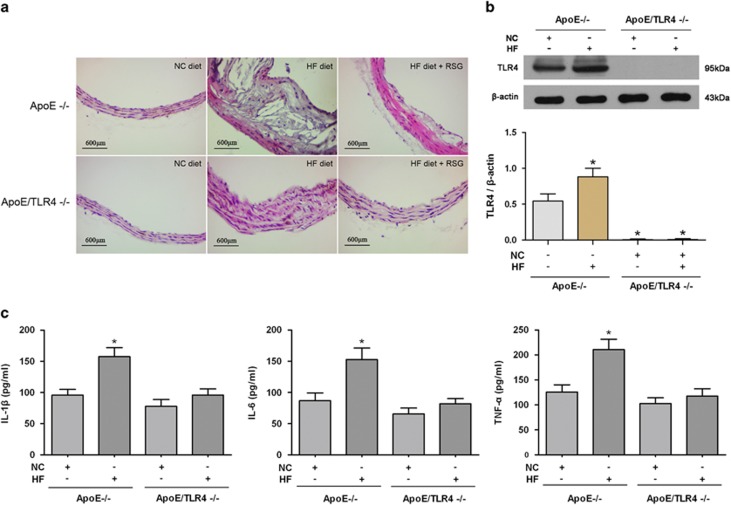
TLR4-mediated inflammation is required in atherosclerotic plaque formation. (**a**) Hematoxylin and eosin staining on cross-sections from representative aortas are presented. HF diet significantly induced atherosclerotic plaque formation in ApoE^−/−^ mice. ApoE/TLR4^−/−^ mice only displayed intimal hyperplasia in response to HF diet. RSG inhibited the HF diet-induced atherosclerotic plaque formation in ApoE^−/−^ mice but not in ApoE/TLR4^−/−^ mice. (**b** and **c**) Expression of TLR4 and proinflammatory cytokines (IL-1*β*, IL-6 and TNF-*α*) in aortas were detected by western blot and ELISA. HF diet induced TLR4 expression, and elevated the level of IL-1*β*, IL-6 and TNF-*α* in ApoE^−/−^ mice but not in ApoE/TLR4^−/−^ mice (*P<0.05 *versus* ApoE^−/−^ mice with NC diet). Results were presented as mean±S.D. (error bars) of three independent experiments

TLR4-mediated inflammation was previously reported to participate in the pathogenesis of atherosclerosis.^9, 12^ In the present study, we found that HF diet significantly accelerated the formation of atherosclerotic plaque in ApoE^−/−^ mice but not in ApoE/TLR4^−/−^ mice, although significant intimal hyperplasia was presented in ApoE/TLR4^−/−^ mice (Figure 2a). Meanwhile, HF diet increased the expression of TLR4 and proinflammatory cytokines, including interleukin-1*β* (IL-1*β*), IL-6 and tumor necrosis factor-*α* (TNF-*α*) in ApoE^−/−^ mice. In contrast, HF diet failed to induce the expression of TLR4 and proinflammatory cytokines in ApoE/TLR4^−/−^ mice (Figures 2b and c). These data suggest that TLR4-mediated inflammation has an important role in HF diet-induced atherosclerotic plaque formation.

**Figure 3:**
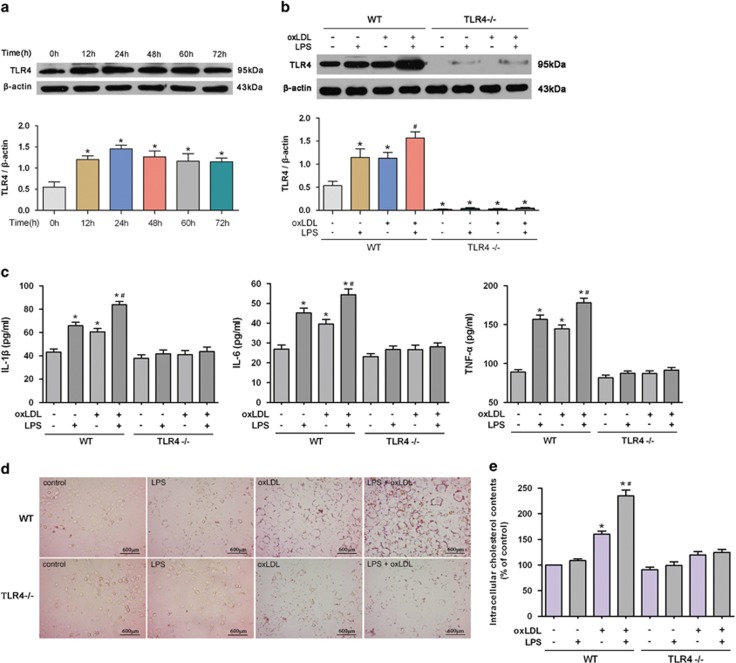
TLR4-mediated inflammation is required in oxLDL-induced VSMC foam cell formation. (**a**) Primary VSMCs from WT mice were incubated with oxLDL (80 *μ*g/ml) for different times (0, 12, 24, 48, 60 or 72 h). TLR4 level was increased in a time-dependent manner, with an obvious effect at 24 h after oxLDL challenge (*P<0.05 *versus* 0 h). (**b**–**e**) Primary VSMCs from WT and TLR4^−/−^ mice were treated with oxLDL and/or LPS (100 ng/ml) for 24 h. OxLDL significantly increased the levels of TLR4 (**b**) and proinflammatory cytokines (IL-1*β*, IL-6 and TNF-*α*) (**c**) in VSMCs from WT mice, which were further elevated by LPS. In contrast, oxLDL and LPS failed to induce the expression of TLR4 (**b**) and proinflammatory cytokines (**c**) in VSMCs from TLR4^−/−^ mice. LPS markedly increased oxLDL-induced lipid droplet accumulation (**d**) and intracellular cholesterol elevation (**e**) in VSMCs from WT mice. By contrast, oxLDL and LPS failed to significantly increase lipid droplet accumulation (**d**) and intracellular cholesterol level (**e**) in VSMCs from TLR4^−/−^ mice (*P<0.05 *versus* control WT-VSMCs; ^#^*P*<0.05 *versus* WT-VSMCs with oxLDL challenge). Results were presented as mean±S.D. (error bars) of three independent experiments

We next examined whether the foam cell formation was affected by TLR4-mediated inflammation in VSMCs. As shown in Figure 3a, exposure to oxLDL upregulated the TLR4 expression in a time-dependent manner, with the maximum induction reached at 24 h. OxLDL-induced TLR4 level was slightly less than the LPS-induced effect, and oxLDL together with LPS synergistically increased the TLR4 expression (Figure 3b). Consistent with the TLR4 level, oxLDL significantly increased the expression of proinflammatory cytokines including IL-1*β*, IL-6 and TNF-*α*, which were further enhanced by LPS (Figure 3c). These data indicate that oxLDL-induced VSMC foam cell formation is accompanied by the activation of TLR4-mediated inflammation.

Using VSMCs from TLR4^−/−^ mice and TLR4 agonist LPS, we next detected the impact of TLR4 on VSMC foam cell formation. TLR4 activation by LPS further increased the oxLDL-induced lipid droplet accumulation (Figure 3d) and intracellular cholesterol elevation (Figure 3e) in VSMCs from wild-type (WT) mice, suggesting that TLR4-mediated inflammation promoted VSMC foam cell formation. However, in VSMCs from TLR4^−/−^ mice, oxLDL and LPS failed to significantly increase lipid droplet accumulation and intracellular cholesterol level (Figures 3d and e), as well as the proinflammatory cytokines (Figure 3c), suggesting that TLR4-mediated inflammation is required in the process of VSMC foam cell formation.

### TLR4 accelerates atherosclerotic plaque formation and VSMC foam cell formation by upregulating the ACAT1 expression

**Figure 4:**
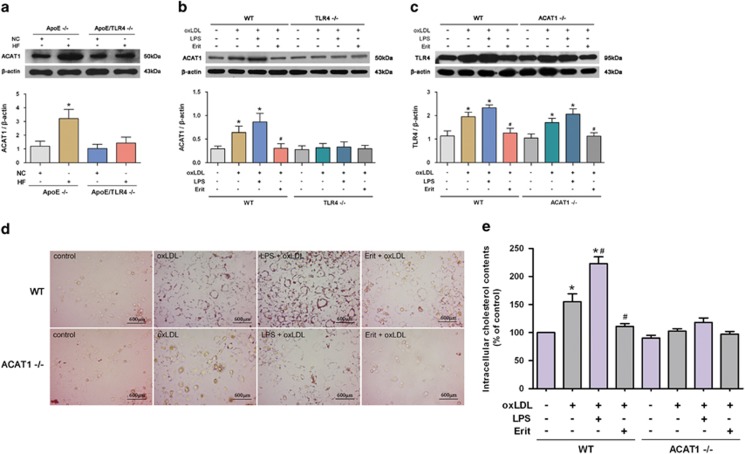
TLR4 accelerates atherosclerotic plaque formation and VSMC foam cell formation by upregulating the ACAT1 expression. (**a**) ACAT1 expression in aortas detected by western blot. HF diet induced ACAT1 expression in ApoE^−/−^ mice but not in ApoE/TLR4^−/−^ mice (*P<0.05 *versus* ApoE^−/−^ mice with NC diet). (**b**) Primary VSMCs from WT and TLR4^−/−^ mice were treated with oxLDL (80 *μ*g/ml) for 24 h in the presence of LPS (100 ng/ml) or eritoran (Erit) (10 ng/ml). OxLDL significantly increased the level of ACAT1 in VSMCs from WT mice. LPS further increased, whereas eritoran significantly impeded, the oxLDL-induced ACAT1 expression. In contrast, VSMCs from TLR4^−/−^ mice failed to regulate the ACAT1 expression in response to oxLDL, LPS or eritoran exposure (*P<0.05 *versus* control WT-VSMCs; ^#^*P*<0.05 *versus* WT-VSMCs with oxLDL challenge). (**c**–**e**) Primary VSMCs from WT and ACAT1^−/−^ mice were treated with oxLDL for 24 h in the presence of LPS or eritoran. OxLDL significantly increased the level of TLR4 in VSMCs from WT and ACAT1^−/−^ mice, which were reverted by eritoran and further enhanced by LPS (**c**). LPS significantly increased oxLDL-induced lipid droplet accumulation (**d**) and intracellular cholesterol elevation (**e**) in VSMCs from WT mice, whereas eritoran exposure exerted the opposite effect. In contrast, oxLDL failed to increase lipid droplet accumulation (**d**) and intracellular cholesterol level (**e**) in VSMCs from ACAT1^−/−^ mice. Neither LPS nor eritoran exerted detectable impact on lipid droplet accumulation (**d**) and intracellular cholesterol level (**e**) in VSMCs from ACAT1^−/−^ mice (*P<0.05 *versus* control WT-VSMCs; ^#^*P*<0.05 *versus* control WT-VSMCs with oxLDL challenge). Results were presented as mean±S.D. (error bars) of three independent experiments

To investigate the association between TLR4 and ACAT1, we examined whether the expression of ACAT1 was increased in a TLR4-dependent manner in ApoE^−/−^ mice fed with an HF diet. As shown in Figure 4a, ACAT1 expression increased markedly in the aortas of ApoE^−/−^ mice fed with an HF diet, and this effect was abolished in ApoE/TLR4^−/−^ mice. These results indicate that HF diet induced atherosclerotic plaque formation via a mechanism involving TLR4-dependent ACAT1 gene expression.

We next manipulated TLR4 using LPS and eritoran *in vitro*. It was found that ACAT1 expression in VSMCs from WT mice was induced significantly by oxLDL. LPS further increased, whereas eritoran impeded, the oxLDL-induced ACAT1 expression. However, VSMCs from TLR4^−/−^ mice failed to upregulate the ACAT1 expression in response to oxLDL or LPS exposure (Figure 4b). These data showed that TLR4 activation increased, whereas TLR4 deficiency impeded, the oxLDL-induced ACAT1, suggesting that TLR4 may accelerate VSMC foam cell formation by upregulating the ACAT1 expression.

To further address this notion, foam cell formation was detected in VSMCs with ACAT1 deficiency and TLR4 manipulation. As shown in Figures 4d and e, activation and inhibition of TLR4, respectively, promoted and suppressed the oxLDL-induced foam cell formation in VSMCs from WT mice. However, ACAT1 deficiency diminished oxLDL-induced VSMC foam cell formation. TLR4 manipulation, no matter activation or inhibition, exerted no detectable impact on foam cell formation in ACAT1 deficiency VSMCs. These findings further suggest that ACAT1 participates in TLR4-regulated VSMC foam cell formation, and TLR4 may promote VSMC foam cell formation by upregulating the ACAT1 expression. On the contrary, ACAT1 deficiency did not affect the TLR4 expression in VSMCs, as shown in Figure 4c.

### TLR4 upregulates ACAT1 expression via MyD88/NF-*κ*B signaling pathway

**Figure 5:**
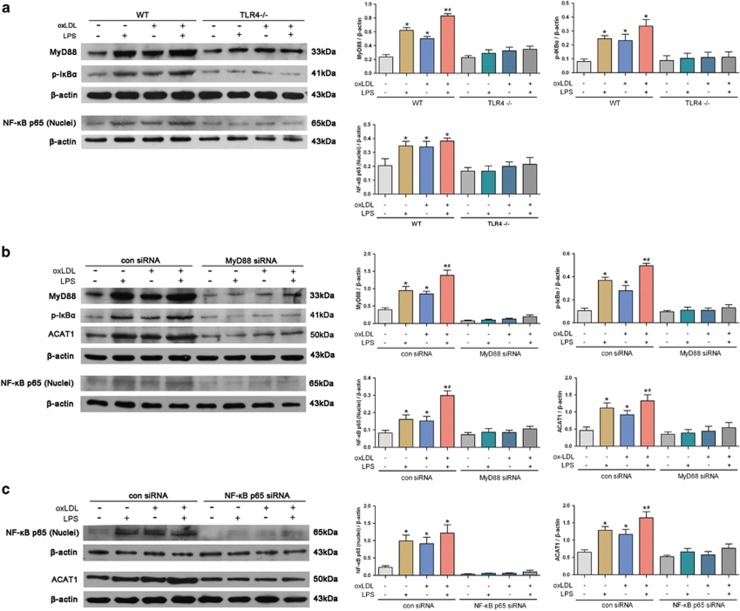
TLR4 upregulates ACAT1 expression via MyD88/NF-*κ*B signaling pathway. (**a**) Primary VSMCs from WT and TLR4^−/−^ mice were treated with oxLDL (80 *μ*g/ml) and/or LPS (100 ng/ml) for 24 h. OxLDL significantly increased the levels of MyD88, NF-*κ*B p65 (nuclei) and p-I*κ*B*α* in VSMCs from WT mice, which similar to LPS-induced effect, and exposure to oxLDL together with LPS, had a synergistic promoting effect. In contrast, oxLDL and LPS failed to induce the MyD88, NF-*κ*B p65 (nuclei) and p-I*κ*B*α* expressions in VSMCs from TLR4^−/−^ mice (*P<0.05 *versus* control WT-VSMCs; ^#^*P*<0.05 *versus* WT-VSMCs with oxLDL challenge). (**b** and **c**) Primary VSMCs from WT mice were transfected with con siRNA, MyD88 siRNA or NF-*κ*B p65 siRNA, and then treated with oxLDL and/or LPS for 24 h. In VSMCs transfected with con siRNA, oxLDL significantly increased the levels of NF-*κ*B p65 (nuclei) (**b**), p-I*κ*B*α* (**b**) and ACAT1 (**b** and **c**), which similar to LPS-induced effect, and exposure to oxLDL together with LPS, had a synergistic promoting effect. In contrast, oxLDL and LPS failed to induce the MyD88, NF-*κ*B p65 (nuclei), p-I*κ*B*α* and ACAT1 expressions in VSMCs transfected with MyD88 siRNA (**b**). oxLDL and LPS also failed to induce the NF-*κ*B p65 (nuclei) and ACAT1 expressions in VSMCs transfected with NF-*κ*B p65 siRNA (**c**) (*P<0.05 *versus* control con siRNA VSMCs; ^#^*P*<0.05 *versus* con siRNA VSMCs with oxLDL challenge). Results were presented as mean±S.D. (error bars) of three independent experiments

It is known that myeloid-differentiating factor 88 (MyD88) and nuclear factor-*κ*B (NF-*κ*B) are the downstream effectors of TLR4 and regulate the expression of many inflammatory genes.^13^ We therefore analyzed whether ACAT1 induction by TLR4 is associated with MyD88 and NF-*κ*B activations. As shown in Figure 5a, oxLDL and/or LPS treatments markedly increased MyD88, NF-*κ*B p65 (nuclei) and phosphorylated I*κ*B*α* (p-I*κ*B*α*) levels in VSMCs from WT mice but not TLR4^−/−^ mice. To further identify the involvements of MyD88 and NF-*κ*B in ACAT1 activation, we used small interfering RNA (siRNA) transfection to, respectively, knock down MyD88 and NF-*κ*B p65 (Figures 5b and c). As expected, knockdown of MyD88 abrogated oxLDL- and LPS-induced expressions of NF-*κ*B p65 (nuclei), p-I*κ*B*α* and ACAT1 (Figure 5b). Moreover, knockdown of NF-*κ*B p65 also impaired oxLDL- and LPS-induced expression of ACAT1 (Figure 5c). These findings indicate that MyD88 and NF-*κ*B mediate TLR4-induced ACAT1 expression, and activation of TLR4/MyD88/NF-*κ*B signaling promotes ACAT1 expression and foam cell formation in oxLDL-loaded VSMCs.

### PPAR*γ* inhibits atherosclerotic plaque formation and VSMC foam cell formation by suppressing TLR4-mediated inflammation and ACAT1 expression

**Figure 6:**
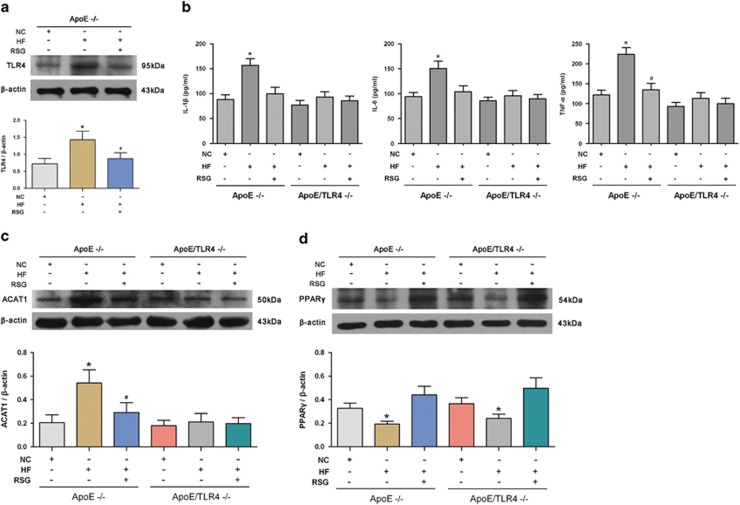
PPAR*γ* inhibits atherosclerotic plaque formation by suppressing TLR4-mediated inflammation and ACAT1 expression. (**a**–**c**) HF diet significantly induced TLR4, proinflammatory cytokines and ACAT1 expressions in ApoE^−/−^ mice, which were significantly abrogated by the PPAR*γ* agonist, RSG. In contrast, ApoE/TLR4^−/−^ mice displayed an undetectable effect on proinflammatory cytokines and ACAT1 in response to RSG. (**d**) HF diet significantly inhibited the level of PPAR*γ* in ApoE^−/−^ mice, which were reversed by RSG. TLR4 deficiency exerted undetectable influence on the expression of PPAR*γ in vivo* (*P<0.05 *versus* ApoE^−/−^ mice with NC diet; ^#^*P*<0.05 *versus* ApoE^−/−^ mice with HF diet). Results were presented as mean±S.D. (error bars) of three independent experiments

Peroxisome proliferator-activated receptor *γ* (PPAR*γ*) has been reportedly involved in the development of atherosclerosis.^14^ Herein, we tested the effect of PPAR*γ* on atherosclerotic plaque formation and the potential roles of TLR4 and ACAT1 in this process. Rosiglitazone (RSG) was used to activate PPAR*γ in vivo*. It was found that RSG significantly impeded the atherosclerotic plaque formation following an HF diet in ApoE^−/−^ mice, but exerted no obvious impact on the plaque formation in ApoE/TLR4^−/−^ mice (Figure 2a). Meanwhile, we found that the elevated expression of TLR4 and proinflammatory cytokines induced by HF diet in ApoE^−/−^ mice was significantly abrogated by the PPAR*γ* agonist, RSG. The same inhibitiory effect of RSG was also observed in ACAT1 expression in ApoE^−/−^ mice fed with an HF diet. In contrast, ApoE/TLR4^−/−^ mice displayed an undetectable effect on proinflammatory cytokines and ACAT1 in response to RSG (Figures 6a–c). Moreover, TLR4 deficiency did not affect the PPAR*γ* expression *in vivo*, as shown in Figure 6d. These data indicate that improving PPAR*γ* expression with RSG inhibited atherosclerotic plaque formation induced by HF diet.

**Figure 7:**
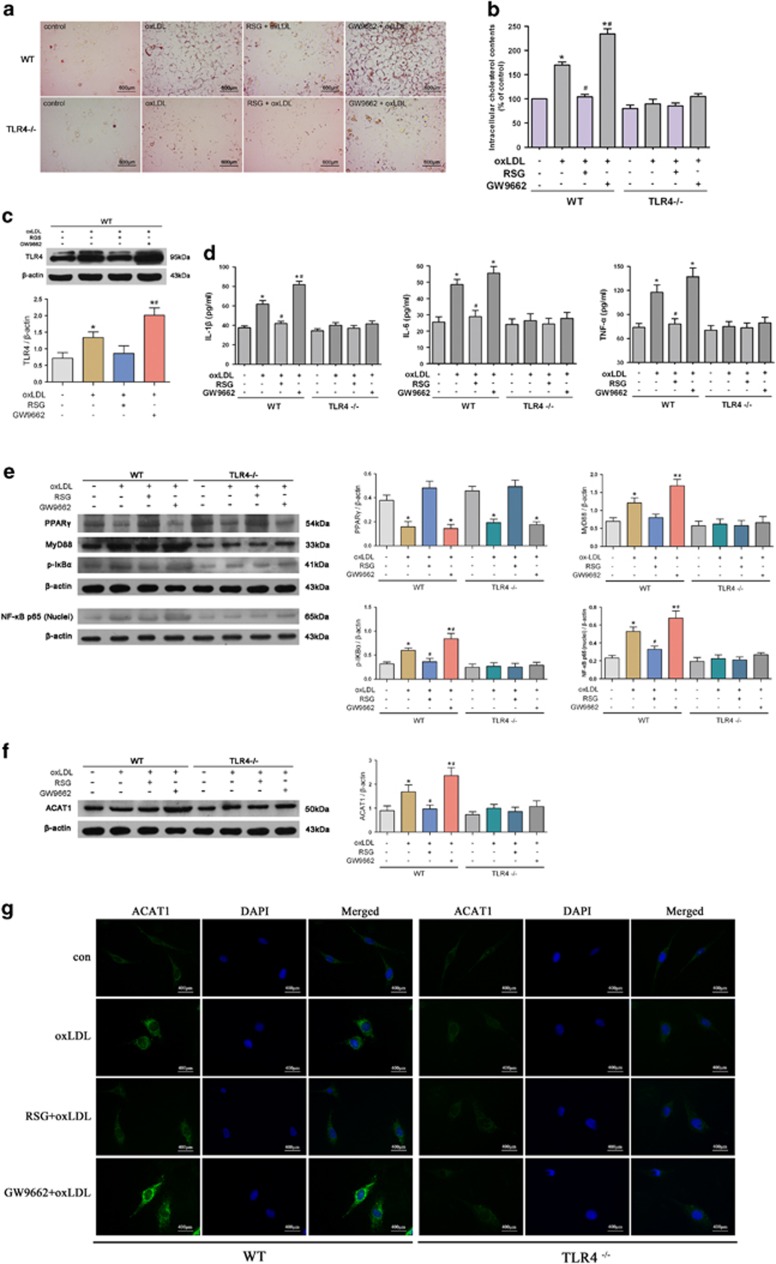
PPAR*γ* inhibits VSMC foam cell formation by suppressing TLR4-mediated inflammation and ACAT1 expression. (**a**–**g**) Primary VSMCs from WT and TLR4^−/−^ mice were treated with oxLDL (80 *μ*g/ml) for 24 h in the presence of RSG (50 *μ*M) or GW9662 (10 *μ*M). RSG significantly inhibited the oxLDL-induced lipid droplet accumulation (**a**) and intracellular cholesterol elevation (**b**) in VSMCs from WT mice, whereas GW9662 exposure exerted the opposite effect. In contrast, oxLDL failed to increase lipid droplet accumulation (**a**) and intracellular cholesterol level (**b**) in VSMCs from TLR4^−/−^ mice. Neither RSG nor GW9662 exerted detectable impact on lipid droplet accumulation (**a**) and intracellular cholesterol level (**b**) in VSMCs from TLR4^−/−^ mice. RSG significantly inhibited, whereas GW9662 further promoted, the oxLDL-induced TLR4 (**c**) and proinflammatory cytokine (**d**) expressions. The same effect of PPAR*γ* was also observed in MyD88, NF-*κ*B p65 (nuclei) and p-I*κ*B*α* expressions in oxLDL-loaded VSMCs (**e**). In contrast, RSG or GW9662 exposure exerted no effect on the expressions of MyD88, NF-*κ*B p65 (nuclei), p-I*κ*B*α* and proinflammatory cytokines in VSMCs from TLR4^−/−^ mice. RSG and GW9662, respectively, suppressed and promoted the oxLDL-induced ACAT1 expression in VSMCs from WT mice, but it was not the case in VSMCs from TLR4^−/−^ mice (**f** and **g**) (*P<0.05 *versus* control WT-VSMCs; ^#^*P*<0.05 *versus* WT-VSMCs with oxLDL challenge). Results were presented as mean±S.D. (error bars) of three independent experiments

Next, we observed the effect of manipulated PPAR*γ* on VSMC foam cell formation *in vitro*. It was found that PPAR*γ* agonist RSG significantly inhibited, whereas PPAR*γ* antagonist GW9662 further promoted, the oxLDL-induced lipid droplet accumulation and intracellular cholesterol elevation. Neither RSG nor GW9662 exerted a detectable effect on foam cell formation in VSMCs from TLR4^−/−^ mice. These data suggest that PPAR*γ* exerts inhibitory effect on VSMC foam cell formation by suppressing TLR4 activation (Figures 7a and b).

Besides, PPAR*γ* activation counteracted the oxLDL-induced inflammation identified by declined TLR4 and proinflammatory cytokines levels, which were further increased by PPAR*γ* inhibition. The same effect of PPAR*γ* was also observed in MyD88, NF-*κ*B p65 (nuclei) and p-I*κ*B*α* expression in oxLDL-loaded VSMCs. In contrast, neither RSG nor GW9662 exerted a detectable effect on TLR4-mediated inflammation in VSMCs from TLR4^−/−^ mice (Figures 7c–e). In agreement with the *in vivo* effect, TLR4 deficiency also exerted an undetectable influence on the expression of PPAR*γ* in VSMCs. (Figure 7e). These data suggest that PPAR*γ* may inhibit VSMC foam cell formation by downregulating the TLR4/MyD88/NF-*κ*B inflammatory signaling in oxLDL-loaded VSMCs.

We next detected the effect of PPAR*γ* on oxLDL-induced ACAT1 expression. It was found that PPAR*γ* activation by RSG significantly inhibited, whereas PPAR*γ* inhibition by GW9662 further promoted, the oxLDL-induced ACAT1 expression in VSMCs from WT mice. However, in VSMCs from TLR4^−/−^ mice, PPAR*γ* manipulation, no matter activation or inhibition, exerted no detectable impact on ACAT1 expression, suggesting that PPAR*γ* exerts inhibitory effect on oxLDL-induced ACAT1 by suppressing TLR4 (Figures 7f and g). Collectively, these data suggest that PPAR*γ* inhibits VSMC foam cell formation by suppressing TLR4-mediated inflammation and ACAT1 expression.

## Discussion

Foam cell formation in the arterial wall is a hallmark of atherosclerosis.^15, 16^ In the later stages of the disease, foam cells undergo apoptosis and secondary necrosis, which causes atherosclerotic plaque rupture, ultimately leading to serious cardiovascular events.^17, 18^ In advanced atherosclerosis lesions, only 30% of foam cells displayed macrophage markers, whereas 45% have a VSMC phenotype.^19^ However, so far the precise mechanisms underlying VSMC foam cell formation have not been well elucidated.

Foam cell formation involves multiple complicated processes including lipid intake, intracellular cholesterol esterification and cholesterol efflux.^16, 20, 21^ ACAT1 is a key and exclusive enzyme involved in intracellular cholesterol esterification, which catalyzes the formation of cholesteryl esters from free cholesterol and stores cholesteryl esters as lipid droplets.^3, 4, 5^ Accumulative evidence supports that cholesteryl esters derived from ACAT1 accumulate within macrophages and VSMCs and results in foam cell formation, which contributes to atherosclerotic plaque formation.^22^ In support of this notion, our study showed that HF diet-induced atherosclerotic plaque formation was accompanied by the upregulation of ACAT1, and that ACAT1 deficiency impaired HF diet-induced effect. In agreement with the *in vivo* findings, oxLDL-induced VSMC foam cell formation was also accompanied by the increased expression of ACAT1. Overexpression of ACAT1 promoted the oxLDL-induced VSMC foam cell formation, whereas ACAT1 deficiency almost completely impeded the oxLDL-induced VSMC foam cell formation. These results suggest that ACAT1 is required in oxLDL-induced VSMC foam cell formation.

Most recently, some mechanisms that involved in foam cell formation have been reported to participate in the regulation of ACAT1 expression. For example, He *et al.*^11^ found that *Chlamydia pneumoniae* upregulated the ACAT1 expression in low-density lipoprotein-loaded macrophage and thus promoted the foam cell formation. Considering that the outer membrane of *Chlamydia pneumoniae* contains the TLR4 agonist LPS, their findings indicate that TLR4-mediated inflammation is related to ACAT1 expression and macrophage-derived foam cell formation.

Unlike the macrophages, VSMCs do not have the inflammatory properties normally. However, in many atherogenic conditions, inflammatory reaction often appears in VSMCs and exerts important roles.^9, 23^ We have demonstrated that TLR4-mediated inflammation appeared in both VSMCs within neointima and cultured VSMCs stimulated by platelet-derived growth factor.^9^ OxLDL, a well-known atherogenic factor, can induce TLR4-mediated inflammatory cytokine expression in cultured VSMCs.^10^ Although still not fully elucidated, the role of inflammation in lipid homeostasis in VSMCs has attracted increasing attention. In particular, the potential role of TLR4-mediated inflammation in oxLDL-induced VSMC foam cell formation and in VSMC and ACAT1 expression needs to be further determined.

To address these questions, we used TLR4^−/−^ mice to clarify the role of TLR4-mediated inflammation in VSMC foam cell formation. We found that HF diet induced atherosclerotic plaque formation and elevated the expression of TLR4 and proinflammatory cytokines, which was consistent with the *in vitro* findings that activating TLR4 by LPS promoted oxLDL-induced VSMC foam cell formation and an inflammatory reaction. TLR4 deficiency inhibited HF diet-induced atherosclerotic plaque formation and impaired VSMC foam cell formation in response to LPS and oxLDL. These findings further demonstrate the close relationship between intracellular inflammation and lipid metabolism disorder in VSMCs. TLR4-mediated inflammation is induced by lipid stimulation, and *vice versa*, activated inflammation exerts essential role in the process of foam cell formation in VSMCs.

Regarding the molecules that involved in TLR4-modulated VSMC foam cell formation, we particularly focused on the role of ACAT1. As mentioned above, *Chlamydia pneumoniae* promoted the macrophage foam cell formation by upregulating ACAT1 expression,^11^ which highlighted the role of TLR4 in ACAT1 regulation. Higashimori *et al.*^24^ found that TLR4 deficiency in VSMCs inhibited free cholesterol-induced ACAT1 expression and foam cell formation. Consistently, in the present study, we found that TLR4 activation increased, whereas TLR4 inhibition impeded, the oxLDL-induced ACAT1 expression. In addition, ACAT1 deficiency diminished the effect of TLR4 on VSMC foam cell formation observed above. These data suggest that ACAT1 mediates the effect of TLR4 on VSMC foam cell formation. TLR4 increases the lipid droplet accumulation and intracellular cholesterol level by upregulating the ACAT1 expression, and ultimately promotes the foam cell formation in oxLDL-challenged VSMCs.

In an effort to clarify the signaling pathway downstream of TLR4 that mediates the ACAT1 modulation and foam cell formation in VSMCs, we tested the role of MyD88/NF-*κ*B signaling in this process. MyD88 and NF-*κ*B are the downstream effectors of TLR4,^13^ which regulate the expression of many inflammatory genes and participate in the development of diseases, including cancer,^25^ brain injury,^26^ inflammatory bowel disease and atherosclerosis.^27, 28, 29^ In the present study, the expression of MyD88, NF-*κ*B p65 (nuclei) and p-I*κ*B*α* were induced by oxLDL in VSMCs from WT mice, which was impaired markedly in VSMCs from TLR4^−/−^ mice, suggesting that MyD88/NF-*κ*B signaling exerts important role downstream of TLR4 in inflammation during VSMC foam cell formation. Furthermore, the increased ACAT1 expression in VSMCs by oxLDL and LPS was markedly diminished by MyD88 or NF-*κ*B p65 silencing. Taken together, these findings suggest that TLR4 upregulates ACAT1 expression by activating MyD88/NF-*κ*B signaling pathway and triggering the inflammation, and ultimately promotes VSMC foam cell formation.

PPAR*γ* is an important transcription factor that regulates a large number of genes that are involved in glucose and lipid metabolism.^30, 31^ We have reported that PPAR*γ* inhibited LPS-induced expression of TLR4 and inflammatory cytokines in VSMCs.^9^ A previous study showed that PPAR*γ* inhibition upregulated ACAT1 expression and promoted foam cell formation in LDL-stimulated macrophage.^11^ In the present study, we tested the effect of PPAR*γ* on TLR4 and ACAT1 expression during the process of VSMC foam cell formation. Our data demonstrated the inhibitory effect of PPAR*γ* on atherosclerotic plaque formation, accompanied by reduced expression of TLR4, proinflammatory cytokines and ACAT1 accordingly. Further study showed that PPAR*γ* also exerted significantly inhibitory effect on foam cell formation and TLR4/MyD88/NF-*κ*B inflammatory signaling in oxLDL-loaded VSMCs. The expression of ACAT1 induced by oxLDL was also suppressed by PPAR*γ*. Interestingly, the above-mentioned effect of PPAR*γ* were largely diminished in TLR4^−/−^ mice and VSMCs. Taken together, these data suggest that PPAR*γ* exerts inhibitory effect on ACAT1 expression by suppressing TLR4/MyD88/NF-*κ*B signaling pathway, and eventually inhibits the VSMC foam cell formation and atherosclerotic plaque formation.

The crosstalk between lipid homeostasis and inflammation has been increasingly investigated.^32, 33, 34^ For example, atherosclerotic lesion, characterized by the disorder of lipid homeostasis, is also a chronic inflammatory process.^33^ Interestingly, this crosstalk also occurs within the cells, especially in the process of macrophage foam cell formation. In the present study, we provide evidence that TLR4/MyD88/NF-*κ*B inflammatory signaling is activated by oxLDL in VSMCs, which in turn upregulates the ACAT1 expression and finally contributes to VSMC foam cell formation. Herein, our findings demonstrate the essential role of TLR4-mediated inflammation in foam cell formation in VSMC, a normally non-inflammatory cell type, and thus provide further insight into the mechanisms of VSMC foam cell formation.

## Materials and Methods

### Reagents

Cell culture reagents were purchased from Gibco-BRL (Carlsbad, CA, USA). OxLDL was purchased from AbD Serotec (Oxford, UK). LPS (TLR4 ligand; *Escherichia* coli 055 : B5) was from Sigma-Aldrich (St. Louis, MO, USA). TLR4 inhibitor, eritoran (E5564), was obtained from Eisai Inc. (Andover, MA, USA). RSG was supplied by Cayman (Ann Arbor, MI, USA). PPAR*γ* antagonist GW9662 was purchased from Sigma-Aldrich. Lipofectamine 2000 was from Invitrogen (Carlsbad, CA, USA). Antibodies targeting ACAT1, TLR4, MyD88, NF-*κ*B p65, p-I*κ*B*α*, PPAR*γ* and *β*-actin, MyD88 siRNA (sc-35986) and NF-*κ*B p65 siRNA (sc-29410) were purchased from Santa Cruz Biotechnology (Santa Cruz, CA, USA).

### Animal care

C57BL/6J WT mice, ApoE^−/−^, TLR4^−/−^ and ACAT1^−/−^ mice, 8–10 weeks of age, were purchased from the Jackson Laboratory (Bar Harbor, ME, USA). The mice were weaned at 4 weeks of age, and received nomal chow (NC) diet containing 5% fat. Atherosclerosis was induced by feeding of 8-week-old ApoE^−/−^, ApoE/TLR4^−/−^ and ApoE/ACAT1^−/−^ mice (*n*=6 mice per group) with an HF diet containing 0.2% cholesterol and providing 42% calories as fat for 8 weeks. To determine the effects of the PPAR*γ* agonist RSG on atherosclerotic plaque formation, RSG (10 mg/kg body weight) was administered intragastrically for 8 weeks starting from the day of HF diet. All the mice were maintained on a controlled light cycle schedule of 12 : 12 h (light/dark) at 25 °C with food and water *ad libitum*. Animal care and procedures conformed with the Guide for the Care and Use of Laboratory Animals. Protocol approval was obtained from the Animal Research Committee of the Third Military Medical University.

### Histopathology

Mice fed an HF diet for 8 weeks were killed, blood serum was collected and aortas were perfusion fixed *in situ* with 4% paraformaldehyde (pH 7.4) and then harvested. Perfusion-fixed arterial segments were embedded in paraffin, and cut transversely into 5-*μ*m-thick sections. Thereafter, the sections were used for hematoxylin and eosin staining. All the required reagents were obtained from Lab Vision (Fremont, CA, USA). Images were recorded by the TissueGnostics microscope (Zeiss, Oberkochen, Germany).

### Cell culture

VSMCs were isolated from the thoracic aorta of 6- to 8-week-old male WT, TLR4^−/−^ or ACAT1^−/−^ mice using a previously described method.^35^ Briefly, all the mice were killed by neck breaking. Then, the thoracic aortic was quickly removed under aseptic conditions and immediately rinsed with aseptic phosphate-buffered saline (PBS) containing 100 U/ml penicillin and 100 mg/ml streptomycin. After stripping the endothelium and adventitia, the aortic media were cut into 1 × 1 mm^2^ small pieces. The pieces were cultured in Dulbecco' s modified Eagle's medium (DMEM) containing 20% fetal bovine serum (FBS), 100 U/ml penicillin and 100 mg/ml streptomycin at 37 °C in an incubator containing 95% air and 5% CO_2_. When the cells formed a confluent monolayer (10–14 days), they were passaged and maintained in the growth medium (DMEM containing 10% FBS, 100 U/ml penicillin and 100 mg/ml streptomycin). The cultured VSMCs were verified by positive immunofluorescence for smooth muscle-specific *α*-actin. Second- to sixth-generation cells were selected for the experiments.

To inhibit MyD88 and NF-*κ*B expressions, the siRNA transfections were performed using Lipofectamine 2000 according to the manufacturer's instruction. A non-related scrambled siRNA was used as a negative control (con siRNA). ACAT1 overexpression was produced by transduction of cells with adenoviral vectors containing mouse ACAT1 cDNA (ACAT1-ov). Adenovirus-expressing ACAT1 was generated using the ViraPower Adenoviral Expression System (Invitrogen) and transfected into the cultured VSMCs for 24 h. Further experiments were performed after 48 h of transfection. The silencing or adenovirus-expressing efficiency was measured by western blot analysis.

### Oil Red O staining for foam cell

Cultured VSMCs were plated on six-well plates and treated with targeted reagents for 24 h in serum-free DMEM. Afterwards, the cells were washed two times with PBS, fixed for 20 min in 4% paraformaldehyde and stained for 30 min in 0.3% Oil Red O. The cells were then washed three times with PBS and photographed with a microscope at × 400 magnification.

### Quantitation of intracellular cholesterol content

Intracellular total cholesterol content was detected according to the method reported earlier by Xue *et al.*^36^ In brief, VSMCs after oxLDL treatment were collected into a centrifuge tube and intracellular lipids were extracted by adding 100 *μ*l of isopropylalcohol. After sonification, the mixtures were centrifuged for 10 min at 1500 ×  *g*. Then, the supernatant was collected for detecting intracellular cholesterol by performing an enzymatic assay. Meanwhile, total protein concentration was detected by analyzing the sediment using the Bradford assay method. The results were then expressed in microgram of cholesterol per milligram of cellular protein.

### Western blot analysis

Protein samples were obtained either from homogenized arteries or cultured cells, and the protein concentration was determined. Protein samples (40 *μ*g) were separated using 10% sodium dodecyl sulfate-polyacrylamide gel electrophoresis and transferred to polyvinylidene fluoride membranes. The membranes were blocked for 2 h in TBS containing 0.05% Tween-20 (TBST) and 5% nonfat milk powder. Then, the membranes were incubated overnight at 4 °C with primary antibodies against ACAT1 (1 : 1000), TLR4 (1 : 1000), MyD88 (1 : 1000), NF-*κ*B (p65) (1 : 1000), p-I*κ*B*α* (1 : 1000), PPAR*γ* (1 : 1000) and *β*-actin (1 : 2000). After extensive washing in TBST, the membranes were incubated with secondary antibodies for 2 h at room temperature. Proteins were visualized with the enhanced chemiluminescence (ECL) Kit (Thermo Scientific, Waltham, MA, USA) and quantified using Labwork 4.6 (UVP, Upland, CA, USA). Density measurements were then normalized to *β*-actin readings.

### Enzyme-linked immunosorbent sssay

The blood serum and supernatant of primary VSMCs were collected, and levels of IL-1*β*, IL-6 and TNF-*α* were quantified using commercially available Enzyme-linked Immunosorbent Assay (ELISA) Kits (R&D Systems, Minneapolis, MN, USA). All assays were performed according to the manufacturer's instructions.

### Immunofluorescence

Cultured VSMCs were fixed with 4% paraformaldehyde and permeablized with 0.1% Triton X-100. Nonspecific proteins were then blocked with 1% bovine serum albumin at room temperature. After blocking, samples were incubated with primary antibody at the dilution of 1 : 100 overnight at 4 °C. FITC-conjugated secondary antibodies (1 : 100 dilution) was used to detect the primary antigen–antibody reaction. Nuclei were stained with 10 mg/ml DAPI (4′-6-diamidino-2-phenylindole; Serva, Heidelberg, Germany) for 5 min at room temperature. Immunofluorescent labeling of the sections was observed with a fluorescence microscope (Nikon Eclipse 55i; Nikon, Tokyo, Japan).

### Statistical analysis

Data are presented as mean±S.D. of at least three independent experiments. Statistical differences between groups were analyzed by ANOVA test. Statistics were calculated with the GraphPad Prism 5 software package (GraphPad, La Jolla, CA, USA). Differences were considered statistically significant at *P*<0.05.

## Abbreviations

ACAT1: acyl-coenzyme A:cholesterol acyltransferase 1
IL-1*β*: interleukin-1*β*
IL-6: interleukin-6
LPS: lipopolysaccharide
MyD88: myeloid-differentiating factor 88
NF-*κ*B: nuclear factor-*κ*B
oxLDL: oxidized low-density lipoprotein
p-I*κ*B*α*: phosphorylated I*κ*B*α*
PPAR*γ*: peroxisome proliferator-activated receptor *γ*
RSG: rosiglitazone
TLR4: Toll-like receptor 4
TNF-*α*: tumor necrosis factor *α*
VSMC: vascular smooth muscle cell
